# Routine immunization community surveys as a tool for guiding program implementation in Kaduna state, Nigeria 2015–2016

**DOI:** 10.1186/s12889-018-6197-8

**Published:** 2018-12-13

**Authors:** Terna I. Nomhwange, Faisal Shuaib, Fiona Braka, Sambo Godwin, Usman Kariko, Umeh Gregory, Sisay G. Tegegne, Bassey Okposen, Chima Onoka

**Affiliations:** 1 World Health Organization Country Office, Abuja, Nigeria; 2 National Primary Health Care Agency, Abuja, Nigeria; 3 World Health Organization, Kaduna Field Office, Kaduna, Nigeria; 4 Kaduna State Primary Healthcare Development Agency, Kaduna, Nigeria

**Keywords:** Routine immunization, Community survey, Immunization status, Kaduna

## Abstract

**Background:**

Routine childhood immunization remains an important strategy for achieving polio eradication and maintaining a polio-free world. To address gaps in reported administrative coverage data, community surveys were conducted to verify coverage, and guide strategic interventions for improved coverage.

**Methods:**

We reviewed the conduct of community surveys by World Health Organization (WHO) field volunteers deployed as part of the surge capacity to Kaduna state and the use of survey results between July 2015 and June 2016. Monthly and quarterly collation and use of these data to guide the deployment of various interventions aimed at strengthening routine immunization in the state.

**Results:**

Over 97,000 children aged 0–11 months were surveyed by 138 field volunteers across 237 of the 255 wards in Kaduna state. Fully or appropriately immunized children increased from 67% in the fourth quarter of 2015 to 76% by the end of the second quarter of 2016. Within the period reviewed, the number of local government areas with < 80% coverage reduced from eight to zero.

**Conclusions:**

The routine conduct of community surveys by volunteers to inform interventions has shown an improvement in the vaccination status of children 0–11 months in Kaduna state and remains a useful tool in addressing administrative data quality issues.

## Background

Childhood Immunization is a critical cost effective strategy used globally to reduce infant and under 5-year-old mortality rates [1]. In a bid to achieve Sustainable Development Goal (SDG) Goal 3 and related targets, countries have made commitments to addressing access issues with the clamor for Universal Health Coverage [2–4]. The decade of vaccines vision, as described in strategic documents like the Global Vaccine Action Plan (GVAP), lists objectives and recommendations towards improving health by 2020 and beyond, through providing the full benefits of immunization to all [5]. The economic benefits of vaccines are even more critical in Low and Middle-Income Countries (LIMC) with increasing competing priorities with the need to introduce new vaccines [6, 7].

Nigeria marked 2 years without a case of paralytic Wild Polio Virus (WPV) until the recent isolation of three cases in Borno state in 2016 as it prepared for a post-polio immunization system and its recent removal and consequent reversal from the list of polio-endemic countries [8, 9]. To achieve this, a robust system that will deliver routine vaccines through various routine immunization strategies remains critical [10]. The Nigeria expert review committee (ERC) on polio and routine immunization, at its 32nd meeting, held on the 19th and 20th of June 2016 in Abuja, highlighted the need to invest in strengthening routine immunization through a comprehensive primary health care strategy [11]. Various such interventions are ongoing in the country with a focus on 107 identified high-risk Local Government Areas (LGAs) with significant improvement in coverage [12]. Some of these efforts are hampered by challenges in measuring impact using administrative data [13]. Multiple intermittent immunization surveys conducted across the country show differences between administrative data and survey outcomes [14, 15].

Kaduna state, located in northwest Nigeria is one of the 11 high-risk states for polio transmission and has recently enacted a primary health care under one roof (PHCUOR) law and established the state primary health care agency with a primary mandate to improve primary health care services.

A total of 1094 health facilities provide routine immunization (RI) services to a targeted population of 326,118 infants; delivering all vaccines based on the national immunization schedule. The Nigeria Demographic Health Survey (NDHS) 2013 reported routine immunization coverage of 43.7% for the third dose of Diphtheria-Pertussis-Tetanus (DPT) vaccine (now replaced by the pentavalent vaccine) and 44.4% for Oral Polio Vaccine (OPV3) in Kaduna state [16]. Data quality and gaps in the information management has been a challenge affecting the routinely reported administrative data [17].

Guidelines have been reviewed with strategies proposed to address the impact of data quality on program implementation with guidance documented in the country multiyear plan (cMYP) 2016–2020 [18]. Immunization surveys to validate administrative data have been advocated for, and various surveys conducted showed wide variation between local government areas (LGA) and state reported administrative data [10]. The use of community surveys to address gaps and strengthen administrative data management have been proposed as well as considerations of alternatives for measuring routine immunization performance [19].

Kaduna state has over the years experienced various degrees of outbreaks of measles and cerebrospinal meningitis (CSM) despite very high administrative coverage data of routine antigens. In line with the surge in health worker capacity of WHO in Nigeria, personnel and field volunteers have been deployed to various communities.These personnel supervise RI services and conduct surveys of children aged 0–11 monthly using standard program tools and checklists and document vaccination status at sub-district levels to be used to guide and support program interventions.

This study reviews the surveys conducted across Kaduna state involving more than 90,000 children between 2015 and 2016 and its use to guide the implementation of various RI interventions at LGA and state levels. It also reports on survey documentation as a best practice regarding polio legacy and its use in transition planning [20].

## Methods

### Outline of the study design

We conducted a retrospective study to review the trends of community surveys conducted across various communities in Kaduna state across four quarters between July 2015 and June 2016 of children < 1 year of age who were fully or appropriately immunized for age in Kaduna state.

Quantitative data showing various vaccination status variables including the number of settlements and wards was analyzed, and we also reviewed the routine immunization administrative coverage data reported by LGA and state within the same period.

### Subjects

A total of 138 field volunteers had been deployed across 255 wards of the 23 LGAs of Kaduna state based on the World Health Organization (WHO) Nigeria surge capacity to improve program implementation. These levels of staff were mandated, as documented in their terms of reference (TOR), to conduct a minimum of one community survey per month in any settlement in the catchment area of the health facilities in the ward of assignment using a community survey checklist. The adherence to this task was ensured by the WHO accountability framework and quarterly administrative action from the Office of the WHO Representative (WR) in Nigeria with all assigned responsibilities accessed accordingly [21].

A total of 9557 surveys involving 96,597 children aged 0–11 months were conducted across the 23 LGAs between July 2015 and June 2016.The sample size for each survey was 10 children per selected settlement was based on the standard protocol of the WHO Nigeria field volunteer’s checklist and field guide for staff.

All survey data with complete information for 10 children sampled with complete information on all variables as shown on the field volunteer’s checklist. Children in households where mothers were not at home at the time of visits, consent not given by caregivers or selected households with children aged greater than 12 months or incomplete documentation on the checklist were discarded. The study utilized the weekly submissions of field volunteer survey checklists which was transferred to a simple Microsoft Excel® template for collation with the capture of details showing the date of survey, settlement name, ward and classification of children according to vaccination status. This was done monthly at the LGA level and quarterly in the state. 

### Measurements

Data was measured in proportions of vaccination status of all children surveyed and also displayed as simple quarterly trends to show the pattern of various vaccination statuses.

The average number of settlements and wards per quarter were documented. A review of routine immunization administrative coverage data for first and third doses of Pentavalent and oral polio vaccine (OPV) was also shown at LGA and state levels.

### Procedure

All designated WHO staff at quarterly program review meeting were re-sensitized and reoriented on the process for community surveys using the field volunteer’s checklist. The identified template for collation and capture of surveys was also discussed and shared with all LGA facilitators (first line supervisors of the field supervisors) for monthly collation and timelines for quarterly submission to the state level monitoring and evaluation (M&E) Officer. The template included various validation checks to reduce entry errors as well as automatically generated graphs to show simple analysis of vaccination status by ward and LGA.

Surveys were conducted by the staff after community entries and permission by village gatekeepers, mainly the village head and consent by caregiver sort and given, in selected households to participate in the survey. Vaccination status was documented using vaccination cards with an assessment of vaccination status for the current age of child based on the current EPI schedule for Nigeria.

Sampling technique for surveys included skipping 1 or 2 households in settlements with less than 10 or greater than 20 Households respectively. A selected household without an eligible child was skipped and the next household with a child sampled.

Collated data were reviewed and proportions of various vaccination status category including “fully or appropriately immunized,” “partially immunized” and “not immunized” and shared at the state emergency operations center (sEoC) and comparison with administrative data. LGA and ward survey reports were reviewed and interventions best suited deployed. These interventions deployed based on best fit are outlined in Table 1.

**Table 1:** Special Interventions deployed in Kaduna state to address areas with low proportions of fully or appropriately immunized children

Thematic Area	Intervention	Target	Expected Outcome
Service Delivery	Expanded Outreach Services	Communities far from Health Center (>5Kms)	Increase Immunization coverage
Service Delivery	Transit Point Vaccination	Mobile Population and child at risk of being missed	Increase vaccination coverage
Service Delivery	Health Camps	Under 5 children and Mothers	Treatment of minor ailments, deworming, health promotion
Service Delivery	Maternal, Neonatal and Child Health (MNCH) week	Pregnant mothers and children less than 5 years	Improved Maternal and Child/Neonatal Care
Service Delivery	Mobile Health Teams	Hard to reach Population (Distance, geography, financial)	Increase health outcomes for at-risk populations
Communication & Social Mobilization	Newborn referrals	Mothers of Babies born at home	Increased neonatal health
Communication & Social Mobilization	Defaulter Tracking	Caregivers or mothers of children who have missed an immunization appointment	Reduction in Vaccination drop-out rates
Vaccine stock management	Vaccine dashboard	Health facilities	Reduction of vaccines stock out reports
Data managements	Monitoring/Distribution of vaccine data tools/Child Cards	Health Facilities	Increased data quality and Documentation of services provided
Vaccine Delivery	Vaccine push	Health Facility	Availability of vaccines at all sessions

At the LGA level, these survey reports were shared with the local immunization officer, director of health and displayed at the government office by ward conspicuously. This data set was also discussed at the monthly review meetings with routine immunization providers for all health facilities in the LGA with clear directives on action points to address issues in a settlement with low levels of appropriately immunized children including supportive supervision.

## Results

A total of 97,007children aged 0-11 months were surveyed between July 2015 and June 2016 by 138 field level staff in Kaduna state (Table 2). These surveys were conducted in a number of wards ranging from 222 to 246 across the four quarters under review involving an average of 24,252 children per quarter.

**Table 2:** Summary of Children showing number of settlements and wards surveyed by field staff in Kaduna state July 2015–June 2016

	Settlements	Wards	No of Children Surveyed
Q3 2015	2407	241	23,921
Q4 2015	2111	222	21,216
Q1 2016	2517	246	26,221
Q2 2016	2563	240	25,649
Average	2400	237	

**Fig. 1:**
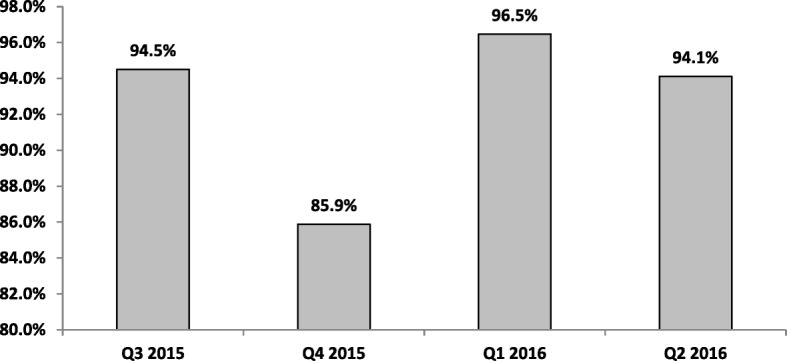
Proportion of wards where surveys were conducted in Kaduna July 2015–June 2016

In total, 97,007children were surveyed as against the expected 16,560 as recommended between July 2015 and June 2016 (Table 3). The highest number of children were surveyed in quarter one of 2016 and the least in quarter 4 of 2015.This survey spread ranged from 241 wards in quarter 3 of 2015 to 240 in quarter 2 of 2016 (Fig. 1) across the 255 wards in Kaduna state with differences in wards surveyed across quarters. An average of 237 Wards and 2400 settlements were surveyed per quarter.

**Table 3:** Distribution of completeness of survey of children aged 0–11 months by Quarter Kaduna July 2015–June 2016

	Expected Children to be survey per Quarter	Reported Children surveyed per quarter
Q3 2015	4140	23,921
Q4 2015	4140	21,216
Q1 2016	4140	26,221
Q2 2016	4140	25,649
Total	16,560	97,007

**Fig. 2:**
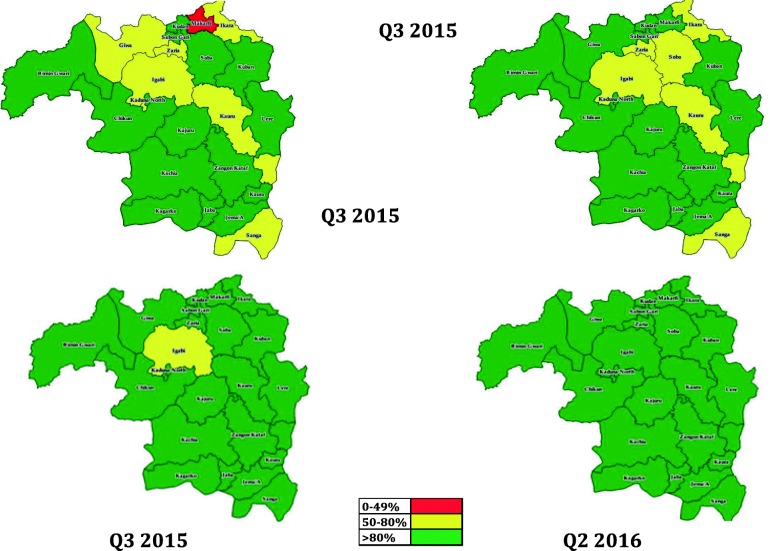
Categorization of LGAs by the third dose of Pentavalent Vaccine Coverage of LGAs in Kaduna state Jan 2015 – June 2016

In 2015, eight LGAs reported Penta 3 coverage < 80% in quarter 1 of 2015, and 6 LGAs in quarter 2 of 2015 reported Penta 3 coverage < 80% (Fig. 2). Only Igabi LGA in quarter 3 2015 had a coverage level of between 50 and 80% while all LGAs in quarter 2 2016 reported coverages of > 80%.

**Fig. 3:**
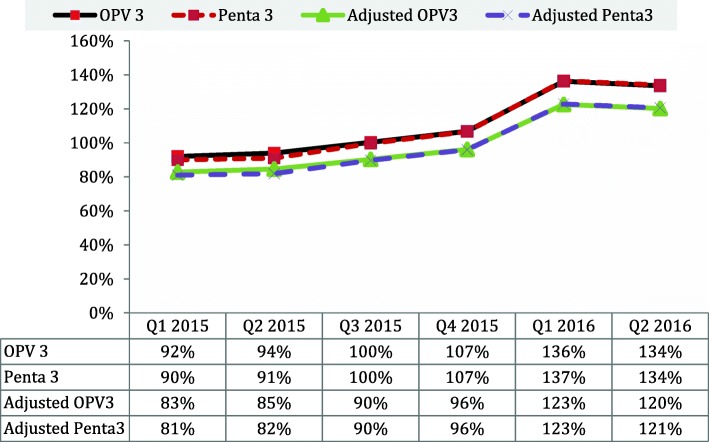
Trend of state Administrative Quarterly RI coverage of Penta3 and OPV3 in Kaduna January 2015–June 2016

The third dose of oral polio vaccine (OPV3) and Penta 3 was 90 and 92% respectively in quater1 2015, OPV3 94% and Penta 3 91% in quater2 2015, 100% in quater3 2015, and 107% in quater4 2016 (Fig. 3). In 2016, the reported administrative coverage reported was 136 and 137% for OPV3 and Penta 3 respectively quarter 1 and 134% each in quarter 2.

The state administrative coverage for the third dose of Penta and OPV coverage trend by quarter shows increases bore both vaccines between 2015 and 2016. (Also adjusted based on data quality audit (DQA) conducted by the state in 2015 with an over-reporting factor of 10%).

**Fig. 4:**
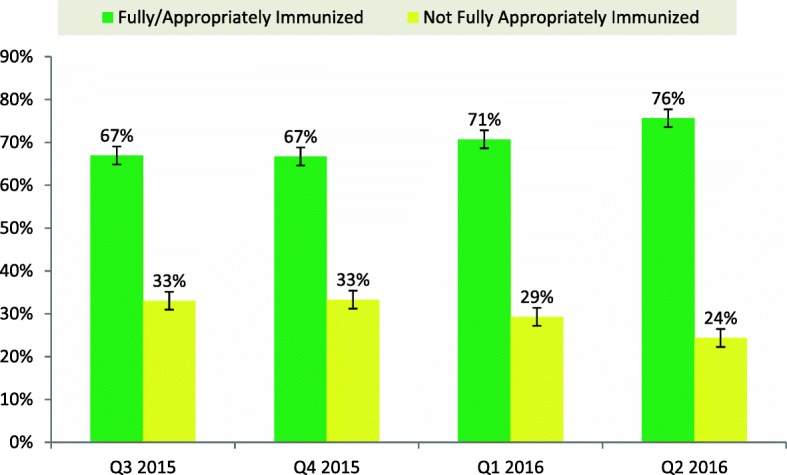
Quarterly Trends of Immunization status from survey of children aged 0-11 months in Kaduna July 2015–June 2016

The summary of children surveyed and their vaccination status was documented across four quarters (Table 4). The proportion of children fully or appropriately immunized was 67% in quarter 3 of 2015 and 76% in quarter 2 2016. The proportion of children not appropriately immunized reduced from 33% in quarter 3 of 2015 to 24% by the end of quarter 2 in 2016 (Fig. 4). This trend is seen with a difference between Fully and not fully immunized children increasing from 34 to 52% (Table 5).

**Table 4:** Distribution showing vaccination status of children aged 0-11 Months from community surveys conducted by field staff in Kaduna state July 2015–June 2016

	Fully/Appropriately Immunized (%)	Partially Immunized (%)	Not Immunized (%)	Total
Q3 2015	16,014(67%)	5897(25%)	2010(8%)	23,921
Q4 2015	14,279(67%)	5291(25%)	1646(8%)	21,216
Q1 2016	18,541(71%)	5165(20%)	2515(10%)	26,221
Q2 2016	19,401(76%)	4712(18%)	1536(6%)	25,649
Total	68,235	21,065	7707	97,007

**Table 5:** Comparison of proportion of vaccination status by quarter July 2015–June 2016

	Fully Immunized	Not Fully Immunized	Variance (%)
Q3 2015	67%	33%	34%
Q4 2015	67%	33%	34%
Q1 2016	71%	29%	42%
Q2 2016	76%	24%	52%

## Discussion

In our reviews of the outputs of the surveys conducted in Kaduna state involving > 90,000 children between July 2015 and June 2016, we found an increasing trend of fully or appropriately immunized children from 67% in quarter 3 2015 to 76% in quarter 2 2016.The use of these routine surveys results to guide various routine immunization intensification activities has also shown to have a positive impact on routine immunization coverage in Kaduna with an increase of Penta 3 coverage data from 90% before the use of these survey data to 134% by the end of June 2016.This result is similar to documented variance with survey data seen to be less than administrative values in South Sudan [22].

The impact was also seen at lower levels of an increase in the number of LGAs with Penta 3 coverage from 65 to 100%. We have also shown a reduction in variance between OPV3 and Penta3 coverage, antigens scheduled at the same hospital visit, a simple method for data quality check. We have also shown increases in surveys conducted in quarter 1 and quarter2 of 2016 compared to those done in 2015. We attribute this to increasing awareness of the importance of the surveys and more interest by the Local authorities’ staff to support the process.

Not all 255 wards in Kaduna state were surveyed within the study period. The survey scope was highest in Q1 2016 and lowest in Q4 2015.This variance in surveys in wards are linked to the decision by field staff to focus on settlements they consider as poor performing regarding routine immunization based on information from the Health facility supervisory visit. We, however, note that some data submissions with quality issues or surveys not done based on guidelines were discarded. Even though we also see variation in numbers of settlements, we have noted that some settlements were surveyed more than once within the period of the survey. These repeat surveys were only conducted in specifically highlighted settlements with clear risks such as disease outbreaks of VPDs or validation visits of interventions already deployed.

Even though a huge number of children were sampled in these surveys, our study was limited by the fact that not all the wards and settlements in the state were surveyed, and surveys were mainly conducted around communities close to a health facility. This limitation is based on the current staff deployment of 138 field officers in the state. This limitation can be addressed by the push for the involvement of by LGA and state Health personnel which could then ensure sure complete state coverage. The limitation of surveys around health facilities is based on transportation cost as the personnel are also not provided with any means of transportation or entitled to transportation reimbursements. With increasing government buy-in and participation, health department vehicles may subsequently be available for the conduct of settlement surveys far from the health facilities but within the reaching every ward (REW) catchment area of the health facility.

This study has shown that routinely administered community of surveys in Kaduna has been used effectively to guide the various routine immunizations intervention activities in the state with a resultant increase in state and LGA level administrative data and with high-level coordination support shown improvement inappropriately immunized children.

We recommend the continuous routine conduct of household surveys by local government, other partner and all field staff at regular intervals and use of vaccination status of children under 1 year to routine immunization activities across Nigeria and other developing countries with administrative data management challenges. These surveys can also be useful in strengthening immunization systems and PHC delivery. Tao W et al. have suggested that reported routine immunization coverage may be lower than reported [23]. Sustaining these interventions that address gaps in data quality remain critical to maintaining high coverage, thus protect children from preventable diseases and death.

## Conclusion

Community surveys are a very effective tool to guide implementation of routine immunization services at community levels with a noticeable increase in proportion of fully immunized children. The use of simple tools to track vaccination status of children and provide regular feedback to program authority at local levels remain critical in the push to reach all eligible children with vaccines and improve coverage.

## Abbreviations

CMYP: Country multiyear plan
CSM: Cerebrospinal meningitis
DPT: Diphtheria Pertussis Tetanus containing vaccine
DQA: Data quality audit
EPI: Expanded programme on immunization
ERC: Expert review committee
GVAP: Global vaccination action plan
LGA: Local government area
LMIC: Low and middle income countries
M&E: Monitoring and evaluation
NDHS: Nigeria demographic health survey
OPV: Oral polio vaccine
PHCUOR: Primary health care under one roof
REW: Reaching every ward
RI: Routine immunization
SDG: Sustainable development goal
SEOC: State emergency operations centre
TOR: Terms of reference
VPD: Vaccine preventable diseases
WHO: World health organization
WPV: Wild polio virus
